# Characterization of the meningococcal DNA glycosylase Fpg involved in base excision repair

**DOI:** 10.1186/1471-2180-9-7

**Published:** 2009-01-09

**Authors:** Katrina L Tibballs, Ole Herman Ambur, Kristian Alfsnes, Håvard Homberset, Stephan A Frye, Tonje Davidsen, Tone Tønjum

**Affiliations:** 1 Centre for Molecular Biology and Neuroscience and Institute of Microbiology, University of Oslo, Rikshospitalet, NO-0027 Oslo, Norway; 2 Institute of Microbiology, Rikshospitalet, NO-0027 Oslo, Norway

## Abstract

**Background:**

*Neisseria meningitidis*, the causative agent of meningococcal disease, is exposed to high levels of reactive oxygen species inside its exclusive human host. The DNA glycosylase Fpg of the base excision repair pathway (BER) is a central player in the correction of oxidative DNA damage. This study aimed at characterizing the meningococcal Fpg and its role in DNA repair.

**Results:**

The deduced *N. meningitidis *Fpg amino acid sequence was highly homologous to other Fpg orthologues, with particularly high conservation of functional domains. As for most *N. meningitidis *DNA repair genes, the *fpg *gene contained a DNA uptake sequence mediating efficient transformation of DNA. The recombinant *N. meningitidis *Fpg protein was over-expressed, purified to homogeneity and assessed for enzymatic activity. *N. meningitidis *Fpg was found to remove 2,6-diamino-4-hydroxy-5-formamidopyrimidine (faPy) lesions and 7,8-dihydro-8-oxo-2'-deoxyguanosine (8oxoG) opposite of C, T and G and to a lesser extent opposite of A. Moreover, the *N. meningitidis fpg *single mutant was only slightly affected in terms of an increase in the frequency of phase variation as compared to a mismatch repair mutant.

**Conclusion:**

Collectively, these findings show that meningococcal Fpg functions are similar to those of prototype Fpg orthologues in other bacterial species.

## Background

*Neisseria meningitidis*, or the meningococcus (Mc), exclusively colonizes the oro- and nasopharynx of humans. It resides as a commensal in approximately 10% of healthy individuals [1], but may become virulent, disseminating into the bloodstream and crossing the blood-brain barrier [2]. Mc septicaemia and meningitis are the cause of significant morbidity and mortality worldwide [2].

On the mucosal surface of the upper respiratory tract, Mc is exposed to reactive oxygen species (ROS) produced by the immune system through the oxidative burst and by endogenous aerobic metabolism, causing damage to many cellular components, including DNA [3]. Oxidative DNA lesions comprise single- and double strand breaks, abasic (apurinic/apyrimidinic, or AP) sites, and base damages, among which one of the most common is the oxidation product of guanine, 7,8-dihydro-8-oxo-2'-deoxyguanosine (8oxoG). The mutagenic 8oxoG can mispair with adenine during replication and cause G:C → T:A transversions [4]. 2,6-diamino-4-hydroxy-5-formamidopyrimidine (faPy) is another oxidative modified form of guanine that inhibits DNA synthesis [5].

The base excision DNA repair pathway (BER) is the main defense against the mutagenic and cytotoxic effects of endogenously damaged bases. This enzymatic pathway has been identified in all organisms studied to date [6]. A DNA glycosylase initiates this pathway by cleaving the glycosylic bond between its specific base substrate and the sugar-phosphate backbone, leaving an abasic (AP) site [6]. Many DNA glycosylases also have an inherent AP lyase activity that cleaves the sugar-phosphate backbone at the AP site, which is subsequently repaired by further BER enzymes. In *E. coli*, formamidopyrimidine-DNA glycosylase (Fpg) shows substrate specificity for 8oxoG and faPy lesions, and exhibits AP lyase activity, in successive β- and δ-elimination steps, leaving a single strand break [7].

In *E. coli*, the mutagenic effects of oxidated guanines are prevented by a triplet of enzymes termed the GO system [8]. In GO, Fpg acts together with the DNA glycosylase MutY which removes adenine when mispaired with 8oxoG, and MutT, a nucleotide hydrolase that converts 8oxoGTP to 8oxoGMP, preventing incorporation of oxidized GTPs into the genomic DNA. Mc single *fpg *mutants only elicit a weak mutator phenotype [9], however, *mutYfpg *double mutants exhibit a much higher increase in spontaneous mutation frequency than would be expected if *fpg *and *mutY *were involved in unrelated repair mechanisms [9]. This synergistic effect of the two Mc DNA glycosylases confirms their essential role in the repair of oxidative DNA damage and a relationship similar to that in the *E. coli *GO system. *In vivo *Mc Fpg activity has previously been detected in whole cell extracts of clinical isolates by cleavage of 8oxoG opposite C [10], however, the Mc Fpg substrate specificity has not previously been investigated.

In this study, the Mc *fpg *gene was cloned and its gene product over-expressed and purified to homogeneity. Recombinant Mc Fpg was assessed with regard to its enzymatic activity towards recognized Fpg DNA substrates. The Mc MC58 Fpg DNA sequence [11], flanking regions and predicted amino acid sequence was analyzed. Furthermore, sequences of *fpg *homologues and flanking regions in other neisserial species were aligned and examined. Finally, an Mc *fpg *mutant was assessed with regard to phase variation rate and compared to that of the wildtype strain and mismatch repair defective mutants. In essence, the Mc Fpg predicted structure and the activity pattern detected were similar to those of prototype Fpg orthologues in other species.

## Methods

### Bacterial strains, plasmids, and DNA manipulations

Bacterial strains and plasmids used in this study are listed in Table 1. DNA isolation, PCR amplification and cloning were performed according to standard techniques [12]. The *fpg *gene from Mc strain M1080 was PCR amplified using primers KT1b and KT2b (Table 2). The *fpg*-containing DNA fragment was cloned into the expression vector pET22b, creating plasmid pET22b-*fpg*M1080. *E. coli *ER2566 was used for pET22b-*fpg*M1080 plasmid propagation. DNA sequencing of pET22b-*fpg*M1080 was performed on a Beckman Coulter CEQ8000 Genetic Analyser System (Beckman Instruments, Ca) using an ABI BigDye Terminator v. 3.1 DNA sequencing kit (Applied Biosystems, NC), with primers listed in Table 2.

**Table 1:** Bacterial strains and plasmids used in this study.

Plasmid, strains	Relevant characteristic	Source/Reference
**Plasmids**		
pET22b	Expression vector, T7 promoter-driven system, His-tag, amp^R^	Novagen
pET22b-*fpg*M1080	pET22b harbouring *fpg *from Mc M1080	This study
pARR2107	Contains an Universal Rate Of Switching cassette	[22]
pUD	pARR2107 harbouring a 12-mer DUS	This study
**Strains**		
***Escherichia coli***		
ER2566	Expression host with chromosomal copy of the T7	New England
	RNA polymerase gene	Biolabs
ER2566-pET22b-*fpg*M1080	ER2566 expressing Mc M1080 *fpg *from pET22b	This study
***Neisseria meningitidis***		
M1080	Serogroup B, isolated in the USA in 1970	[45]
Z1099	Serogroup A, isolated in the Philippines in 1968	Dominique A. Caugant
NmZ1099_UROS	Z1099 harbouring a Universal Rate Of Switching cassette	This study
NmZ1099_UROSΔ*fpg*	Z1099 *fpg *strain harbouring a Universal Rate Of Switching cassette	This study
NmZ1099_UROSΔ*mutS*	Z1099 *mutS *strain harbouring a Universal Rate Of Switching cassette	This study

**Table 2:** The DNA sequences of primers used in this study.

Oligonucleotide	Sequence (5'-3')*	Application	Source
KT1b	gggaattccatatgcctgaattgccggaagtggaaacg	Cloning	This study
KT2b	cgcgctcgagtttctgacagttcgggcaata	Cloning	This study
TD146	gaagtggaaacgacgttgcgcg	Sequencing	This study
TD147	cgtgccgcgctgccccaaagtttc	Sequencing	This study
TD160	ctcataccaaagtatcgc	Sequencing	This study
TD161	ttcgccccaccgtcctgc	Sequencing	This study
TD46	gctgttggaaaaactggg	Sequencing	This study
TD47	gcatacagataatccgtgc	Sequencing	This study
spcFOR	cccagtggacataagcctgt	G-tract control, PCR/sequencing	[22]
spcREV	agccgaagtttccaaaaggt	G-tract control, PCR/sequencing	[22]
N248	ggcggcatgaccc**8oxog**gaggcccatc	DNA substrate Containing 8oxoG lesion	Eurogen
T248	gatgggcctc**c**gggtcatgccgcc	DNA substrate, complementary to N248	Eurogen
1393	gatgggcctc**a**gggtcatgccgcc	DNA substrate, complementary to N248	Eurogen
1394	gatgggcctc**g**gggtcatgccgcc	DNA substrate, complementary to N248	Eurogen
1395	gatgggcctc**t**gggtcatgccgcc	DNA substrate, complementary to N248	Eurogen
H7	aacaacaacaaatgccgtctgaaccaacatgccgtctgaaaacaacaacaac	Undamaged DNA substrate	This study
H8	gttgttgttgttttcagacggcatgttggttcagacggcatttgttgttgtt	Undamaged DNA substrate, complementary to H7	This study

* letters in bold represents the DNA lesion or its complementary base in the DNA substrate

### Bioinformatics analyses and search for signature motifs

An *in silico *search for functional domains and DNA binding motifs was carried out on the deduced amino acid sequence of Mc MC58 Fpg (NMB1295), using the the Expasy site http://us.expasy.org/cgi-bin/protscale.pl and the PROSITE [13] and Pfam databases [14]. The electrostatic charge was calculated using the EMBOSS package http://emboss.sourceforge.net/. The DNA sequences of the *fpg *genes and flanking regions as well as the deduced amino acid sequences were aligned and compared in a panel of neisserial species for which the genome sequences were available. The following genome sequences (with accession numbers) were downloaded from Genbank http://www.ncbi.nlm.nih.gov/: *Neisseria gonorrhoeae *FA1090 (NC_002946), Mc MC58 serogroup B (NC_03112) [11], Mc Z2491 serogroup A (NC_003116) [15], Mc FAM18 serogroup C (NC_03221) [16] and Mc 053442 serogroup C (NC_010120) [17]. The temporary sequence data for *Neisseria lactamica *ST-640 was obtained from the Pathogen Sequencing Unit at the Sanger Institute ftp://ftp.sanger.ac.uk/pub/pathogens/nl/. Access to the genome sequence of Mc 8013 serogroup C was provided by Eduardo Rocha, ABI/Institut Pasteur, Paris, France, with kind permission from Vladimir Pelicic, Necker Hospital, Paris/Imperial College London. Prediction of the Fpg secondary structure was performed based on a blast search http://www.ncbi.nlm.nih.gov/blast/Blast.cgi in the JPred program [18]. Protein data bank (PDB) structural modeling was performed using SMART http://smart.embl-heidelberg.de/, Pfam http://www.sanger.ac.uk/Software/Pfam/, Phyre http://www.sbg.bio.ic.ac.uk/phyre/ and PyMol http://www.pymol.org.

The Mc *fpg *flanking regions were searched for the presence of transcriptional terminators with the GeSTer genome scanner for terminators (the last version of the program is available at http://pallab.physics.iisc.ernet.in/~sum05/anil/projects.html and the TransTermHP program http://transterm.cbcb.umd.edu/. Operon predictions were taken from VIMSS [19]. Putative promoters were identified with the transcription promoter predictor available at the Berkeley Drosophila Genome Project http://www.fruitfly.org/seq_tools/promoter.html and the BPROM predictor of bacterial promoters http://www.softberry.com/berry.phtml. The gene organization of the *fpg *flanking regions in different species was compared using the String program http://string.embl.de/.

### Purification of the recombinant Mc M1080 Fpg protein

*E. coli *strain ER2566 over-expressing Mc Fpg encoded by the plasmid pET22b-*fpg*M1080 was grown in LB medium containing ampicillin to a final concentration of 100 μg/ml at 37°C with shaking until OD_600 _was 0.6. The cells were induced with 1 mM isopropyl-D-thiogalactopyranoside and grown for 4 hours. Cells were harvested and washed in phosphate-buffered saline and stored at -70°C. The cells were resuspended in sonication buffer containing 50 mM Na_2_HPO_4_/NaH_2_PO_4_, 300 mM NaCl, pH 8.0 and protease inhibitor complete without EDTA (Roche Applied Science, Germany) before lysis by sonication. The cleared lysate was loaded onto a Ni-NTA agarose column (Qiagen, Germany) and the column washed with wash buffer containing 20 mM imidazole. Bound protein was eluted with a step gradient of 2 column volumes of the elution buffer containing 40, 60, 80, 100, 140, 180, 220 and 250 mM imidazole. Fractions containing purified protein were pooled and dialysed against 25 mM Tris-HCl, pH 7.5, 300 mM NaCl and 10% glycerol.

### Assay for base excision of 8oxoG opposite C, A, G or T

Duplex DNA substrates containing a single 8oxoG opposite of C, A, G or T were generated by ^32^P 5' end-labelling of oligonucleotides, using T4 polynucleotide kinase (New England Biolabs, MA) followed by annealing to a complementary oligonucleotide [20]. The oligonucleotide sequences of the DNA substrates are listed in Table 2. DNA glycosylase reactions were performed by mixing purified protein with 10–50 fmol DNA substrate in a total volume of 10 μl. The enzyme activities were assayed in the reaction buffer previously described [20] and incubated at 37°C for 30 min. *E. coli *Fpg (New England Biolabs, MA) was included as a positive control. Products of the reactions were separated by 20% denaturing PAGE and visualized by phosphorimaging. The assay was performed in triplicate.

### Assay for formamidopyrimidine (faPy) DNA glycosylase activity

*N*-[H3]-*N*-methyl-*N*'-nitrosourea (MNU; 1.5 Ci mmol^-1^) was used to prepare poly(dG-dC) DNA (12,000 dpm mg-1) [21]. DNA glycosylase activity was assayed by mixing purified protein with substrate in a reaction buffer containing 70 mM 3-(*N*-morpholino) propane sulfonic acid, pH 7.5, 1 mM EDTA, 1 mM dithiothreitol (DTT) and 5% glycerol for 30 min at 37°C. Removal of bases was measured in a total reaction volume of 50 μl containing 14 μg of DNA substrate and 500 ng of purified meningococcal protein or 160 U of *E. coli *Fpg (New England Biolabs, MA). The assay was repeated 5 times.

### Screening for phase variation by use of a universal rate of switching (UROS) cassette

To promote efficient natural transformation, a 12-mer DNA uptake sequence was inserted into plasmid pARR2107 containing a Universal Rate of Switching (UROS) cassette (kind gift from H. L. Alexander, Emory University School of Medicine, Atlanta, GA) [22], creating plasmid pUD. Mc strain Z1099 (kind gift from D. A. Caugant, Norwegian Institute of Public Health, Oslo, Norway) was transformed with pUD and named NmZ1099_UROS. The *mutS *and *fpg *genes of NmZ1099_UROS were inactivated by insertion of a kanamycin resistance cassette as described by Davidsen *et al*., 2007 [9] in two separate genetic transformations creating strains NmZ1099_UROSΔ*mutS *and NmZ1099_UROSΔ*fpg*. The mononucleotide tract of 8 G residues preceding the spectinomycin resistance gene of the UROS cassette was confirmed as an intact 8-mer by PCR and sequencing (by using the primers listed in Table 2) in all three strains before switching frequency/phase variation was assessed. Briefly, Mc strains were grown overnight at 37°C, 5% CO_2_, before 10 colonies were resuspended in GC broth. Serial dilutions were plated on GC agar with and without spectinomycin (to a final concentration of 50 mg/l) and incubated overnight. The spectinomycin OFF to ON switching rate was determined by dividing the number of colonies on GC plates containing spectinomycin by the number of colonies on plain GC plates. Phase variation experiments were repeated at least 5 times for each strain. Significance in differences in phase variation frequency was calculated by the Kruskal-Wallis test.

## Results and discussion

Fpg is nearly ubiquitous among bacterial species and is highly conserved both within annotated neisserial genome sequences and clinical Mc isolates [10], as well as between evolutionarily distant prokaryotes. We examined the activity and specificity of recombinant Mc Fpg purified to homogeneity towards representative substrates resulting from oxidative DNA damage, 8oxoG and faPy, and detected prototype Fpg glycosylase activity. Previously, we have shown a synergistic effect between the two GO components MutY and Fpg in Mc [9]. Together, these findings emphasize a distinct role for Fpg in the defense against the deleterious effects of reactive oxygen species.

**Figure 1:**
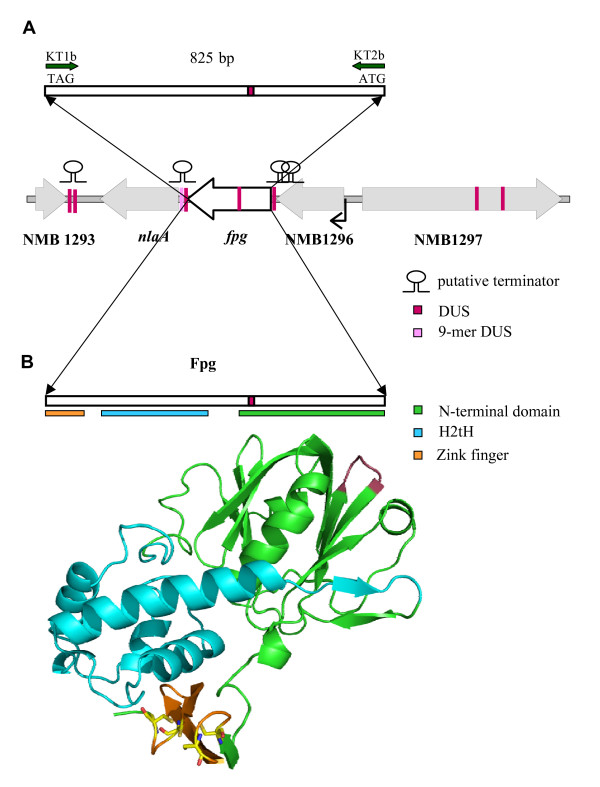
***N. meningitidis *(Mc) Fpg**. (A) Physical map of the Mc *fpg *open reading frame and flanking regions. The *fpg *gene contains a DNA uptake sequence (DUS). Primers KT1b and KT2b employed in cloning of the Mc *fpg *gene are depicted. The gene organization of the Mc *fpg *flanking regions is identical in all available neisserial genomes. NMB1296 encodes a hypothetical protein with sequence homology to DNA methyltransferases. A promoter is predicted upstream of NMB1296 (black arrow). The *fpg *and the lysophophatidic acid acyltransferase *nlaA *genes are putatively co-transcribed [27], although an inverted repeat (containing DUS) associated with transcription termination or attenutation is found downstream of the *fpg *gene. NMB1297 is COG-annotated *mltD *(membrane-bound lytic murein transglycosylase). NMB1293 is a hypothetical protein. The distribution of DUS and degenerate DUS is indicated. (B) Structural modeling of Mc Fpg based on *E. coli *Fpg (PDB 1k82) showing the DNA binding motifs helix-two-turn-helix (H2tH) (blue) and zinc finger (orange), as well as the N-terminal domain (green) containing the glycosylase catalytic amino acid residues. Amino acids encoded by DUS are highlighted in purple.

The putative Mc *fpg *open reading frame (ORF) consists of 828 bp and contains a DNA uptake sequence (DUS) (5'-GCCGTCTGAA-3') (Figure 1A). The Mc genome harbours approximately 2000 copies of this highly conserved 10 bp sequence, which is required for efficient transformation [23]. A 12-mer DUS with two additional bp upstream of the core 10 bp repeat element improves the transformation efficiency [24]. The Mc *fpg *gene contains one 11-mer. A single complete DUS or AT-DUS (10-, 11- or 12-mer) may promote the reacquisition of a gene by transformation if it is damaged or deleted and DUS occurs at higher densities in genome maintenance genes than in other house-keeping genes [25].

The organization of the *fpg *flanking region is unique for *Neisseria *species http://string.embl.de/ (data not shown). Upstream of the *fpg *gene are the hypothetical ORFs NMB1297 and NMB1296 (Figure 1A). NMB1297 is annotated as an ortholog to *mltD *http://www.ncbi.nlm.nih.gov/COG/, which encodes a membrane-bound lytic murein transglycosylase of unknown function. NMB1296 shows 30–40% amino acid identity with DNA methyltransferases in a number of bacterial species http://www.ncbi.nlm.nih.gov/blast/Blast.cgi. Downstream, *fpg *is flanked by the *nlaA *gene, encoding a lysophosphatidic acid acyltransferase involved in biosynthesis of the glycerophosholipid membrane [26], about 300 bp of non-coding sequence containing two DUS within a predicted terminator, and the opposite oriented hypothetical ORF NMB1293. The NMB1296, *fpg *and *nlaA *genes are all oriented in the same direction and a putative promoter is found upstream of NMB1296 while none are identified between these genes. At the end of NMB1296 a terminator is predicted by TransTermHP. Between *fpg *and *nlaA*, a terminator is predicted by GeSTer. This intrinsic terminator contains a DUS and an imperfect DUS as inverted repeat, a structure found in many putative Mc transcription terminators or attenuators [24]. The VIMSS Operon Prediction suggests co-transcription of *fpg *and NMB1296. However, Swartley and Stephens have evidence by reverse transcriptase PCR that *nlaA *and *fpg *are co-transcribed in Mc strain NMB [27]. In microarray analysis of an MC58 *fpg *mutant compared to wildtype, *nlaA *was the only gene significantly down-regulated at least 1.5 fold, supporting the evidence for co-transcription of these two genes (unpublished data).

The Mc *fpg *open reading frame encodes 276 amino acids containing a predicted N-terminal glycosylase catalytic domain, a helix-two-turn-helix and a C-terminal zinc finger (Figure 1B, additional file 1, Figures S1 and S2). These regions contain long sequences with a positive electrostatic charge, enforcing binding to negatively charged DNA (See additional file 1, Figure S3). Alignment of the deduced Fpg sequence from the genomes of five Mc strains reveals non-synonymous or synonymous substitutions in 5 out of 276 amino acid positions (see additional file 1, Figure S1). The positions showing variation correspond exactly to those found in the *fpg *gene from 11 Mc clinical isolates previously sequenced [10]. An additional 6 amino acids show non-synonymous or synonymous variation when the *N. gonorrhoeae *and *N. lactamica *sequences are included in the comparison. All known functional residues exhibit complete sequence conservation (see additional file 1, Table S1 and Figure S1). Comparison of the neisserial Fpg sequences to those in species where the Fpg crystal structure is solved [28-31] also shows a high degree of conservation, especially in the functional domains and catalytic amino acid residues (see additional file 1, Figure S2). This conservation was confirmed by *in silico *fusion of the crystal structure of *Lactococcus lactis *Fpg with Mc Fpg using the PDB (Figure 1B). Interestingly, the 11-mer DUS sequence encodes amino acids that are not identified as functional residues and is localized in an *fpg *region showing relatively low sequence homology across species boundaries (see additional file 1, Figures S1 and S2).

**Figure 2:**
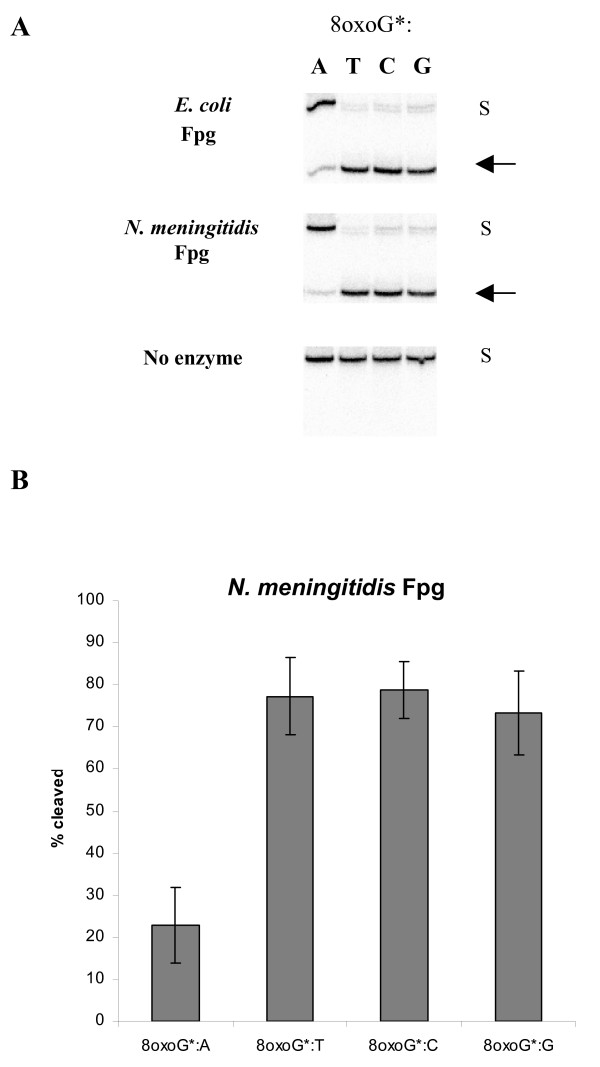
**DNA glycosylase activity of *N. meningtidis *(Mc) recombinant Fpg protein**. (A) 1 ng of purified Mc Fpg or 0.032 Units of *E. coli *Fpg was incubated with 10–50 fmol of a 24 bp duplex oligodeoxyribonucleotide containing a single 8oxoG residue opposite A, T C or G. Base excision and strand cleavage were analysed by 20% PAGE and phosphorimaging. The arrow indicates the cleaved DNA substrate. * denotes ^32^P-labelled strand. S; substrate. (B) Quantification of strand cleavage activity by Mc Fpg. The results represent the average of three independent experiments and error bars indicate the standard deviation of the mean.

Fpg has been extensively studied in *E. coli *and is characterized in several other prokaryotes as well [32-34], displaying identical substrate specificities. In order to analyze the substrate specificity of Mc Fpg, the gene was over-expressed in *E. coli *and recombinant Mc Fpg protein purified to homogeneity (see additional file 1, Figure S4). Mc Fpg has an apparent size in SDS-PAGE of approximately 30 kDa, corresponding to the molecular weight predicted from the genome deduced amino acid sequence and similar to Fpg of *E. coli *and *L. lactis *[32,33]. The preferred substrates for recognized Fpg proteins are 8oxoG and faPy residues. The ability of recombinant Mc Fpg to remove these lesions was investigated, using *E. coli *Fpg as a positive control. Activity towards C:faPy residues in a ^3^H-labeled poly(dG-dC) substrate was identified (Table 3). When assessing the 8oxoG excision, the Mc Fpg displayed both DNA glycosylase and AP lyase activity (Figure 2). Equivalent levels of base excision of 8oxoG opposite C, T and G and much lower activity toward 8oxoG when mispaired with A was demonstrated (Figure 2). No activity was dectected in the absence of 8oxoG residues (see additional file 1, Figure S5). This discrimination of the base opposite the lesion is in keeping with findings on *E. coli *Fpg [35], although the remaining activity against 8oxoG:A seen in Mc Fpg was not found in the original characterization of substrate specificity in *E. coli*. 8oxoG:C is probably the most important physiological substrate for Mc Fpg, despite the similar levels of nicking observed in 8oxoG:T and 8oxoG:G, as the former is by far the most common substrate *in vivo *in *E. coli *[4]. The removal of 8oxoG from the genome prevents G:C→T:A transversions in *E. coli*, but the mutation rates in single *fpg *mutants are too low in Mc to detect these lesions [9], despite this being the most likely event when 8oxoG is preferentially mis-incorporated with adenine and left unrepaired. Recent studies in *M. smegmatis *have identified an alternative pattern of preferential incorporation of guanine opposite 8oxoG, creating G:C→C:G transversions or A:T→C:G transitions in the absence of Fpg [36]. 8oxoG:G and G:C→C:G transversions can also be found in *E. coli *and *S. pombe*, however, they are rare compared to 8oxoG:A events. In conclusion, these results demonstrate that the protein encoded by the Mc *fpg *gene excises base lesions that are typical substrates of other Fpg orthologues and are consistent with this protein being an Fpg DNA glycosylase.

**Table 3:** DNA glycosylase activity of *N. meningitidis *(Mc) recombinant Fpg protein.

Substrate	Released bases (fmol)	
	
	Average	(St. dev.)^c^
*N. meningitidis *Fpg^a^	75	(± 30)
*E. coli *Fpg^b^	64	(± 44)
No enzyme	12	(± 4)

^a ^500 ng of protein was employed in each reaction

^b ^160 Units of protein was employed in each reaction

^c ^standard deviation of the mean

Removal of formamidopyrimidine (faPy) from [3H]-methyl-faPy-poly(dG·dC) DNA by recombinant Mc and *E. coli *Fpg. The results are given as the average of five independent measurements.

**Figure 3:**
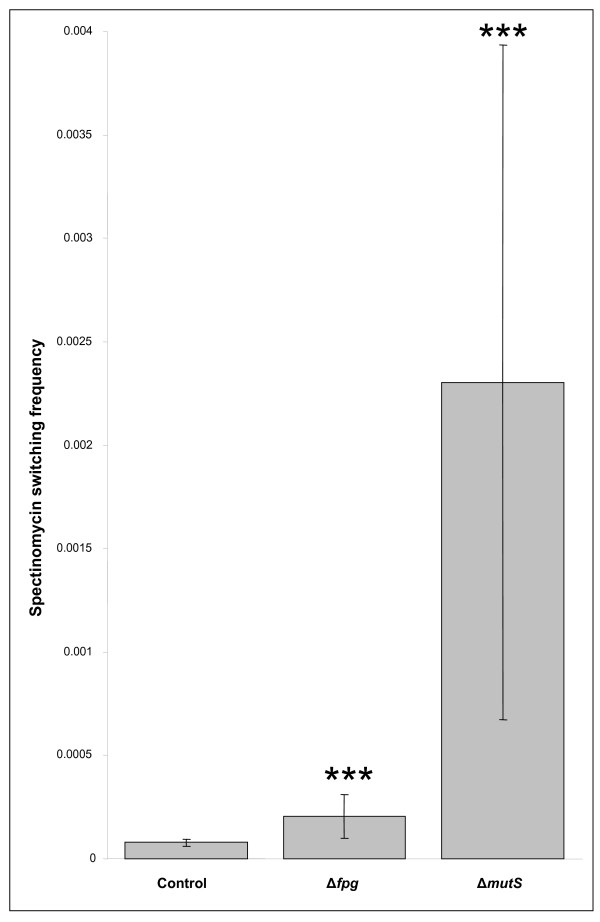
**Assessment of meningococcal (Mc) phase variation**. Phase variation frequency for Mc strains NmZ1099_UROS (Control), NmZ1099_UROSΔ*fpg *(Δ*fpg*) and NmZ1099_UROSΔ*mutS *(Δ*mutS*) as examined by a spectinomycin assay. The results are given as the median of at least 5 independent measurements. Error bars represent ± 1 quartile. Phase variation is moderately and significantly increased, respectively, in the Mc Δ*fpg *(2-fold) and Δ*mutS *(30-fold) background compared to the wild-type level (***p < 0.001).

Mc is a bacterium that seemingly spontaneously produces a plethora of variants upon which selection can act, instead of sensing the environment and changing accordingly [37]. One of the major processes governing genetic changes in *Neisseria sp*. is phase variation. Phase variation is mediated by unstable polynucleotide tracts allowing the gene expression to be switched on or off [37]. Recently, several genome maintenance genes have been shown to modulate phase variation frequencies, including the mismatch repair components *mutS *and *mutL*, the nucleotide excision repair gene *uvrD *and the translesion DNA polymerase *dinB *[38-41]. Since Mc Fpg is able to remove oxidized guanines, although in an error-free manner, we wanted to investigate a potential contribution of Mc *fpg *on phase variation of polyG tracts. Mc strains NmZ1099_UROS (Control), NmZ1099_UROSΔ*fpg *(Δ*fpg*) and NmZ1099_UROSΔ*mutS *(Δ*mutS*) were constructed and examined by S12 ribosomal gene switching in a spectinomycin-selection assay (Figure 3). Phase variation was, as previously reported [38-41], significantly increased in the Δ*mutS *(30-fold) background compared to the wild-type level (***p < 0.001). However, the Mc *fpg *mutant exhibited only moderate increase (2-fold) compared to the wild-type level (***p < 0.001), and thus MutS exerts a more profound effect on the stability of Mc polyG tracts than Fpg. Likewise, the Mc *fpg *mutant was recently shown to generate only a weak mutator phenotype when assessed for its spontaneous mutation frequency in a rifampicin assay [9]. In conclusion, Fpg is not a major player in modulating Mc mutation frequencies.

Although Mc Fpg displays traits characteristic of the Fpg family of proteins, survival rates of a Mc *fpg *mutant were not affected by exposure to reactive oxygen species [9]. This is in contrast to findings in *M. smegmatis*, where H_2_O_2 _exposure proved to be lethal to *fpg *null mutants [36], and in the photosynthetic cyanobacteria *S. elongates *where an *fpg*-deficient strain exhibited progressively reduced survival with increasing levels of oxidatively damaging irradiation [42]. Considering the potential importance of oxidative DNA damage in the Mc habitat combined with the vulnerability of a relatively G+C rich genome obtaining such lesions, the explanation for the species discrepancy should be investigated further. The Fpg family of DNA glycosylases also contains endonuclease VIII (Nei) and eukaryotic Nei orthologues. The Nei proteins excise oxidized pyrimidines and may also serve as a backup for removal of 8oxoG in *E. coli *[43], however, no Mc Nei ortholog has been identified [11,15]. On the other hand, the abundant Mc anti-oxidant system provides particularly high protection towards the generation of such DNA lesions [44]. In general, the elucidation of the Mc DNA repair profile is important for understanding the lifestyle of this important pathogen, commensal and model organism.

## Conclusion

Mc *fpg *contains DUS both within its coding sequence and in close proximity to the open reading frame, potentially promoting reacquisition of this gene by transformation if it is damaged or lost. The *fpg *gene may belong to an operon together with a putative DNA methyltransferase and a lysophosphatidic acid acyltransferase, although the reasons for this gene organisation remain obscure. Both the nucleotide and amino acid sequences of neisserial Fpg homologues are highly conserved. In addition, Mc Fpg amino acid sequence shows great conservation across species boundaries in functional domains, and Mc Fpg contains a predicted N-terminal glycosylase catalytic domain, a helix-two-turn-helix and a C-terminal zinc finger. Accordingly, Mc Fpg exhibits DNA glycosylase and AP lyase activities and remove both 8oxoG and faPy lesions. When examining the stability of polyG tracts, MutS was found to modulate mutation frequencies due to phase variation to a much higher extent than Fpg. In conclusion, Mc Fpg predicted structure and activity pattern were found to be similar to those of prototype Fpg orthologues in other species. Together, these findings emphasize a distinct role for Mc Fpg in the defense against the deleterious effects of reactive oxygen species.

## Authors' contributions

KLT carried out the molecular genetic studies and analysis of purified protein, performed sequence alignments and drafted the manuscript. OHA constructed pUD, designed the phase variation studies and performed the GeSTer analysis. KA contributed to pUD construction and performed the phase variation studies. HH purified recombinant proteins. SAF participated in the bioinformatic analyses. TD supervised the molecular studies and analysis of purified protein, and assisted in manuscript writing. TT conceived the study, participated in its design and coordination and drafted the manuscript. All authors read and approved the final manuscript.

## Supplementary Material

Additional file 1 **Supplementary Material.** contains Table S1 Deduced amino acid sequence of Fpg homologues in Neisseria, Figure S1 Deduced amino acid sequence of Fpg homologues in Neisseria, Figure S2 Deduced amino acid sequence of Fpg orthologues, Figure S3 Electrostatic charge of meningococcal Fpg, Figure S4 Purified meningococcal Fpg, Figure S5 Meningococcal Fpg activity towards undamaged DNA substrate. Click here for file

## References

[B1] Yazdankhah SP Caugant DA *Neisseria meningitidis*: an overview of the carriage state J Med Microbiol 2004 53 821 832 10.1099/jmm.0.45529-0 15314188

[B2] Stephens DS Greenwood B Brandtzaeg P Epidemic meningitis, meningococcaemia, and *Neisseria meningitidis* Lancet 2007 369 2196 2210 10.1016/S0140-6736(07)61016-2 17604802

[B3] O'Rourke EJ Chevalier C Pinto AV Thiberge JM Ielpi L Labigne A Radicella JP Pathogen DNA as target for host-generated oxidative stress: role for repair of bacterial DNA damage in *Helicobacter pylori *colonization Proc Natl Acad Sci USA 2003 100 2789 2794 12601164 10.1073/pnas.0337641100 PMC151419

[B4] Cheng KC Cahill DS Kasai H Nishimura S Loeb LA 8-Hydroxyguanine, an abundant form of oxidative DNA damage, causes G-T and A-C substitutions J Biol Chem 1992 267 166 172 1730583

[B5] Boiteux S Laval J Imidazole open ring 7-methylguanine: an inhibitor of DNA synthesis Biochem Biophys Res Commun 1983 110 552 558 10.1016/0006-291X(83)91185-3 6340667

[B6] Bjelland S Seeberg E Mutagenicity, toxicity and repair of DNA base damage induced by oxidation Mutat Res 2003 531 37 80 14637246 10.1016/j.mrfmmm.2003.07.002

[B7] Bhagwat M Gerlt JA 3'- and 5'-strand cleavage reactions catalyzed by the Fpg protein from *Escherichia coli *occur via successive beta- and delta-elimination mechanisms, respectively Biochemistry (Mosc) 1996 35 659 665 10.1021/bi9522662 8555240

[B8] Michaels ML Miller JH The GO system protects organisms from the mutagenic effect of the spontaneous lesion 8-hydroxyguanine (7,8-dihydro-8-oxoguanine) J Bacteriol 1992 174 6321 6325 1328155 10.1128/jb.174.20.6321-6325.1992 PMC207574

[B9] Davidsen T Tuven HK Bjoras M Rodland EA Tonjum T Genetic interactions of DNA repair pathways in the pathogen *Neisseria meningitidis* J Bacteriol 2007 189 5728 5737 17513474 10.1128/JB.00161-07 PMC1951836

[B10] Davidsen T Amundsen EK Rodland EA Tonjum T DNA repair profiles of disease-associated isolates of *Neisseria meningitidis* FEMS Immunol Med Microbiol 2007 49 243 251 10.1111/j.1574-695X.2006.00195.x 17284282

[B11] Tettelin H Saunders NJ Heidelberg J Jeffries AC Nelson KE Eisen JA Ketchum KA Hood DW Peden JF Dodson RJ Complete genome sequence of *Neisseria meningitidis *serogroup B strain MC58 Science 2000 287 1809 1815 10.1126/science.287.5459.1809 10710307

[B12] Sambrook J Russell DW Molecular cloning: a laboratory manual 2001 Cold Springs Laboratory Press. Cold Spring Harbor, New York

[B13] Hulo N Sigrist CJ Le SV Langendijk-Genevaux PS Bordoli L Gattiker A De CE Bucher P Bairoch A Recent improvements to the PROSITE database Nucleic Acids Res 2004 32 D134 D137 14681377 10.1093/nar/gkh044 PMC308778

[B14] Bateman A Coin L Durbin R Finn RD Hollich V Griffiths-Jones S Khanna A Marshall M Moxon S Sonnhammer EL The Pfam protein families database Nucleic Acids Res 2004 32 D138 D141 14681378 10.1093/nar/gkh121 PMC308855

[B15] Parkhill J Achtman M James KD Bentley SD Churcher C Klee SR Morelli G Basham D Brown D Chillingworth T Complete DNA sequence of a serogroup A strain of *Neisseria meningitidis *Z2491 Nature 2000 404 502 506 10.1038/35006655 10761919

[B16] Bentley SD Vernikos GS Snyder LA Churcher C Arrowsmith C Chillingworth T Cronin A Davis PH Holroyd NE Jagels K Meningococcal genetic variation mechanisms viewed through comparative analysis of serogroup C strain FAM18 PLoS Genet 2007 3 e23 17305430 10.1371/journal.pgen.0030023 PMC1797815

[B17] Peng J Yang L Yang F Yang J Yan Y Nie H Zhang X Xiong Z Jiang Y Cheng F Characterization of ST-4821 complex, a unique *Neisseria meningitidis *clone Genomics 2008 91 78 87 10.1016/j.ygeno.2007.10.004 18031983

[B18] Cuff JA Clamp ME Siddiqui AS Finlay M Barton GJ JPred: a consensus secondary structure prediction server Bioinformatics 1998 14 892 893 10.1093/bioinformatics/14.10.892 9927721

[B19] Price MN Huang KH Alm EJ Arkin AP A novel method for accurate operon predictions in all sequenced prokaryotes Nucleic Acids Res 2005 33 880 892 15701760 10.1093/nar/gki232 PMC549399

[B20] Eide L Bjoras M Pirovano M Alseth I Berdal KG Seeberg E Base excision of oxidative purine and pyrimidine DNA damage in *Saccharomyces cerevisiae *by a DNA glycosylase with sequence similarity to endonuclease III from *Escherichia coli* Proc Natl Acad Sci USA 1996 93 10735 10740 8855249 10.1073/pnas.93.20.10735 PMC38224

[B21] Boiteux S Belleney J Roques BP Laval J Two rotameric forms of open ring 7-methylguanine are present in alkylated polynucleotides Nucleic Acids Res 1984 12 5429 5439 6462910 10.1093/nar/12.13.5429 PMC318929

[B22] Alexander HL Richardson AR Stojiljkovic I Natural transformation and phase variation modulation in *Neisseria meningitidis* Mol Microbiol 2004 52 771 783 10.1111/j.1365-2958.2004.04013.x 15101983

[B23] Goodman SD Scocca JJ Identification and arrangement of the DNA sequence recognized in specific transformation of *Neisseria gonorrhoeae* Proc Natl Acad Sci USA 1988 85 6982 6986 3137581 10.1073/pnas.85.18.6982 PMC282103

[B24] Ambur OH Frye SA Tonjum T New functional identity for the DNA uptake sequence in transformation and its presence in transcriptional terminators J Bacteriol 2007 189 2077 2085 17194793 10.1128/JB.01408-06 PMC1855724

[B25] Davidsen T Rodland EA Lagesen K Seeberg E Rognes T Tonjum T Biased distribution of DNA uptake sequences towards genome maintenance genes Nucleic Acids Res 2004 32 1050 1058 14960717 10.1093/nar/gkh255 PMC373393

[B26] Swartley JS Balthazar JT Coleman J Shafer WM Stephens DS Membrane glycerophospholipid biosynthesis in *Neisseria meningitidis *and *Neisseria gonorrhoeae*: identification, characterization, and mutagenesis of a lysophosphatidic acid acyltransferase Mol Microbiol 1995 18 401 412 10.1111/j.1365-2958.1995.mmi_18030401.x 8748025

[B27] Swartley JS Stephens DS Co-transcription of a homologue of the formamidopyrimidine-DNA glycosylase (*fpg*) and lysophosphatidic acid acyltransferase (*nlaA*) in *Neisseria meningitidis* FEMS Microbiol Lett 1995 134 171 176 8586265 10.1111/j.1574-6968.1995.tb07933.x

[B28] Sugahara M Mikawa T Kumasaka T Yamamoto M Kato R Fukuyama K Inoue Y Kuramitsu S Crystal structure of a repair enzyme of oxidatively damaged DNA, MutM (Fpg), from an extreme thermophile, *Thermus thermophilus *HB8 EMBO J 2000 19 3857 3869 10921868 10.1093/emboj/19.15.3857 PMC306600

[B29] Serre L Pereira de JK Boiteux S Zelwer C Castaing B Crystal structure of the *Lactococcus lactis *formamidopyrimidine-DNA glycosylase bound to an abasic site analogue-containing DNA EMBO J 2002 21 2854 2865 12065399 10.1093/emboj/cdf304 PMC126059

[B30] Gilboa R Zharkov DO Golan G Fernandes AS Gerchman SE Matz E Kycia JH Grollman AP Shoham G Structure of formamidopyrimidine-DNA glycosylase covalently complexed to DNA J Biol Chem 2002 277 19811 19816 10.1074/jbc.M202058200 11912217

[B31] Fromme JC Verdine GL Structural insights into lesion recognition and repair by the bacterial 8-oxoguanine DNA glycosylase MutM Nat Struct Biol 2002 9 544 552 12055620 10.1038/nsb809

[B32] Boiteux S O'Connor TR Lederer F Gouyette A Laval J Homogeneous *Escherichia coli *FPG protein. A DNA glycosylase which excises imidazole ring-opened purines and nicks DNA at apurinic/apyrimidinic sites J Biol Chem 1990 265 3916 3922 1689309

[B33] Duwat P de OR Ehrlich SD Boiteux S Repair of oxidative DNA damage in gram-positive bacteria: the *Lactococcus lactis *Fpg protein Microbiology 1995 141 Pt 2 411 417 7704272 10.1099/13500872-141-2-411

[B34] Senturker S Bauche C Laval J Dizdaroglu M Substrate specificity of *Deinococcus radiodurans *Fpg protein Biochemistry (Mosc) 1999 38 9435 9439 10.1021/bi990680m 10413519

[B35] Tchou J Kasai H Shibutani S Chung MH Laval J Grollman AP Nishimura S 8-oxoguanine (8-hydroxyguanine) DNA glycosylase and its substrate specificity Proc Natl Acad Sci USA 1991 88 4690 4694 2052552 10.1073/pnas.88.11.4690 PMC51731

[B36] Jain R Kumar P Varshney U A distinct role of formamidopyrimidine DNA glycosylase (MutM) in down-regulation of accumulation of G, C mutations and protection against oxidative stress in mycobacteria DNA Repair (Amst) 2007 6 1774 1785 10.1016/j.dnarep.2007.06.009 17698424

[B37] Moxon ER Rainey PB Nowak MA Lenski RE Adaptive evolution of highly mutable loci in pathogenic bacteria Curr Biol 1994 4 24 33 10.1016/S0960-9822(00)00005-1 7922307

[B38] Richardson AR Stojiljkovic I Mismatch repair and the regulation of phase variation in *Neisseria meningitidis* Mol Microbiol 2001 40 645 655 10.1046/j.1365-2958.2001.02408.x 11359570

[B39] Richardson AR Yu Z Popovic T Stojiljkovic I Mutator clones of *Neisseria meningitidis *in epidemic serogroup A disease Proc Natl Acad Sci USA 2002 99 6103 6107 11983903 10.1073/pnas.092568699 PMC122909

[B40] Alexander HL Rasmussen AW Stojiljkovic I Identification of *Neisseria meningitidis *genetic loci involved in the modulation of phase variation frequencies Infect Immun 2004 72 6743 6747 15501815 10.1128/IAI.72.11.6743-6747.2004 PMC522996

[B41] Martin P Sun L Hood DW Moxon ER Involvement of genes of genome maintenance in the regulation of phase variation frequencies in *Neisseria meningitidis* Microbiology 2004 150 3001 3012 10.1099/mic.0.27182-0 15347758

[B42] Muhlenhoff U The FAPY-DNA glycosylase (Fpg) is required for survival of the cyanobacterium *Synechococcus elongatus *under high light irradiance FEMS Microbiol Lett 2000 187 127 132 10856645 10.1111/j.1574-6968.2000.tb09148.x

[B43] Blaisdell JO Hatahet Z Wallace SS A novel role for Escherichia coli endonuclease VIII in prevention of spontaneous G-->T transversions J Bacteriol 1999 181 6396 6402 10515930 10.1128/jb.181.20.6396-6402.1999 PMC103775

[B44] Seib KL Tseng HJ McEwan AG Apicella MA Jennings MP Defenses against oxidative stress in *Neisseria gonorrhoeae *and *Neisseria meningitidis*: distinctive systems for different lifestyles J Infect Dis 2004 190 136 147 10.1086/421299 15195253

[B45] Frasch CE Gotschlich EC An outer membrane protein of *Neisseria meningitidis *group B responsible for serotype specificity J Exp Med 1974 140 87 104 4134571 10.1084/jem.140.1.87 PMC2139694

