# Supplementary material for: Characterization of the meningococcal DNA glycosylase Fpg involved in base excision repair
Source: BMC Microbiol. 2009 Jan 9;9:7. doi: 10.1186/1471-2180-9-7 (PMC2628661; doi:10.1186/1471-2180-9-7)
Supplement: Additional file 1 — Supplementary Material. contains Table S1 Deduced amino acid sequence of Fpg homologues in Neisseria, Figure S1 Deduced amino acid sequence of Fpg homologues in Neisseria, Figure S2 Deduced amino acid sequence of Fpg orthologues, Figure S3 Electrostatic charge of meningococcal Fpg, Figure S4 Purified meningococcal Fpg, Figure S5 Meningococcal Fpg activity towards undamaged DNA substrate. [file 1471-2180-9-7-S1.doc]

Supplementary Material

Characterization of the meningococcal DNA glycosylase Fpg involved in base excision repair

Katrina L Tibballs^1^, Ole Herman Ambur^2^, Kristian Alfsnes^2^, Tonje Davidsen^1^, Håvard Homberset^1^, Stephan A Frye^1,2^ and Tone Tønjum^1,2*^

Email: Katrina L Tibbals – [k.l.tiballs@medisin.uio.no](mailto:k.l.tiballs@medisin.uio.no); Ole Herman Ambur – [ole.herman.ambur@rr-research.no](mailto:ole.herman.ambur@rr-research.no); Kristian Alfsnes – [kristian.alfsnes@rr-research.no](mailto:kristian.alfsnes@rr-research.no); Tonje Davidsen – [tonje.davidsen@rr-research.no](mailto:tonje.davidsen@rr-research.no); Håvard Homberset – [havard.homberset@rr-research.no](mailto:havard.homberset@rr-research.no); Stephan A Frye – [stephan.frye@rr-research.no](mailto:stephan.frye@rr-research.no); Tone Tønjum – [tone.tonjum@rr-research.no](mailto:tone.tonjum@rr-research.no)

^*^Corresponding author.

| **Table S1** | **Deduced amino acid sequence of Fpg homologues in *Neisseria*** | **p 2** |
| --- | --- | --- |
| **Figure S1** | **Deduced amino acid sequence of Fpg homologues in *Neisseria*** | **p 3** |
| **Figure S2** | **Deduced amino acid sequence of Fpg orthologues** | **p 5** |
| **Figure S3** | **Electrostatic charge of meningococcal Fpg** | **p 7** |
| **Figure S4** | **Purified meningococcal Fpg** | **p 8** |
| **Figure S5** | **Meningococcal Fpg activity on undamaged DNA substrate** | **p 9** |
|  | **References** | **p 10** |

**Table S1: Deduced amino acid sequence of Fpg homologues in Neisseria.** Comparison of the deduced amino acid sequences of the Fpg homologue in 7 available *Neisseria* sequences. Essential motifs and catalytic residues, as well as the residues encoded by DNA uptake sequence (DUS) are depicted.

| **Amino acid** | **Characteristic** | **Reference** |
| --- | --- | --- |
| Pro1, Glu2, Glu5, Ile176, Tyr177, Ser221, Thr222, Leu223, Arg224,  Gly230 (putative),  Gly233 (putative) | Specific 8oxoG binding and lesion recognition | [1-18] |
| Met74, Arg114, Phe116 | Insertion into DNA helix, filling gap after removal of base | [8,12,13,16-18] |
| Lys57, His 71, Leu 164, Gly172, Gly174, Asn175, Tyr243, Cys 250, Cys 253, Lys257, Arg263, Cys 270, Cys 273 | Binding of damaged strand of DNA | [4,8,10,12,13,16,19-26] |
| His 95, Lys115 | Binding of complementary strand of DNA | [8,12,18] |
| Arg114 | Recognition of base opposite 8oxoG | [8,12,18] |
| Lys57, Glu138, Lys161, Glu180, Gly230 (putative), Gly233 (putative) | Structural formation essential for glycosylase activity | [8,12,13,15,16,19,20,27] |
| Lys57, Tyr243 | β-elimination | [8,12,13,16,19,20] |
| Tyr243, Arg263 | δ-elimination | [8,12,13,16] |

* 20 * 40 *
Nl_ST64 : MPELPEVETTLRGIAPHIEGKTVEAVVLRQLKLRWQINPDLGEILSGRQV : 50
Ng_FA1090 : MPELPEVETTLRGIAPHIEGKTVEAVILRQLKLRWQINPDLGEILSGRQV : 50
Nm_MC58 : MPELPEVETTLRGIAPHIEGKTVEAVVLRQLKLRWQINPDLGEILSGRQV : 50
Nm_053442 : MPELPEVETTLRGIAPHIEGKTVEAVVLRQLKLRWQINPDLGEILSGRQV : 50
Nm_Z2491 : MPELPEVETTLRGIAPHIEGKTVEAVVLRQLKLRWQINPDLGEILSGRQV : 50
Nm_8013 : MPELPEVETTLRGIAPHIEGKTVEAVVLRQLKLRWQINPDLGEILSGRQV : 50
Nm_FAM18 : MPELPEVETTLRGIAPHIEGKTVEAVVLRQLKLRWQINPDLGEILSGRQV : 50

 60 * 80 * 100
Nl_ST64 : LSCGRRAKYLIVRFQTGILLIHLGMSGSLRIFTPSDGRIGRPDRHDHVDI : 100
Ng_FA1090 : LSCGRRAKYLIVRFQTGILLIHLGMSGSLRIFTPSDGRIGRPDRHDHVDI : 100
Nm_MC58 : LSCGRRAKYLLIRFQTGVLLIHLGMSGSLRIFTPSDGRIGRPDRHDHVDI : 100
Nm_053442 : LSCGRRAKYLIVRFQTGILLIHLGMSGSLRIFTPSDGRIGRPDRHDHVDI : 100
Nm_Z2491 : LSCGRRAKYLIVRFQTGILLIHLGMSGSLRIFTPSDGRIGRSDRHDHVDI : 100
Nm_8013 : LSCGRRAKYLLIRFQTGVLLIHLGMSGSLRIFTPSDGRIGRPDRHDHVDI : 100
Nm_FAM18 : LSCGRRAKYLIVRFQTGILLIHLGMSGSLRIFTPSDGRIGRPDRHDHVDI : 100

 DUS * 120 * 140 *
Nl_ST64 : VFSDGTVMRYRDPRKFGAILWYEGIEEHHPLLEKLGPEPLSEAFCADYLY : 150
Ng_FA1090 : VFSDGTVMRYRDPRKFGAILWYEGIEERHPLLEKLGPEPLSEAFCTDYLY : 150
Nm_MC58 : VFSDGTVMRYRDPRKFGAILWYEGIEEHHPLLEKLGPEPLSEAFCADYLY : 150
Nm_053442 : VFSDGTVMRYRDPRKFGAILWYEGIEEHHPLLEKLGPEPLSEAFCTDYLY : 150
Nm_Z2491 : VFSDGTVMRYRDPRKFGAILWYEGIEEHHPLLEKLGPEPLSEAFCTDYLY : 150
Nm_8013 : VFSDGTVMRYRDPRKFGAILWYEGIEEHHPLLEKLGPEPLSEAFCADYLY : 150
Nm_FAM18 : VFSDGTVMRYRDPRKFGAILWYEGIEEHHPLLEKLGPEPLSEAFCADYLY : 150
 llllllllllllllll
 160 * 180 *
Nl_ST64 : ARLKAQKRAVKLALMDNAVVVGVGNIYANESLFRAGISPHRPANRLKKKE : 200
Ng_FA1090 : AGLKAQKRAVKLALMDNTVVVGVGNIYANESLFRAGISPHRPANRLKKKE : 200
Nm_MC58 : ARLKAQKRAVKLALMDNAVVVGVGNIYANESLFRAGISPHRPANRLKKKE : 200
Nm_053442 : VRLKAQKRAVKLALMDNAVVVGVGNIYANESLFRAGISPHRPANRLKKKE : 200
Nm_Z2491 : VRLKAQKRAVKLALMDNAVVVGVGNIYANESLFRAGISPHRPANRLKKKE : 200
Nm_8013 : ARLKAQKRAVKLALMDNAVVVGVGNIYANESLFRAGISPHRPANRLKKKE : 200
Nm_FAM18 : VRLKAQKRAVKLALMDNAVVVGVGNIYANESLFRAGISPHRPANRLKKKE : 200
 lllllllllllllllllllllH2tHlllllllllllllllllllllllll
 * 220 * 240 *
Nl_ST64 : CALLVETVKAVLQRAIETGGSTLRDFVDSDGKSGYFQQEYTVYGRHNLPC : 250
Ng_FA1090 : CAVLVETVKAVLQRAIETGGSTLRDFVDSDGKSGYFQQEYTVYGRHNQPC : 250
Nm_MC58 : CALLVETVKAVLQRAIETGGSTLRDFVDSDGKSGYFQQEYTVYGRHNQPC : 250
Nm_053442 : CALLVETVKAVLRRAIETGGSTLRDFVDSDGKSGYFQQEYTVYGRHNQPC : 250
Nm_Z2491 : CALLVETVKAVLRRAIETGGSTLRDFVDSDGKSGYFQQEYTVYGRHNQPC : 250
Nm_8013 : CALLVETVKAVLRRAIETGGSTLRDFVDSDGKSGYFQQEYTVYGRHNQPC : 250
Nm_FAM18 : CALLVETVKAVLRRAIETGGSTLRDFVDSDGKSGYFQQEYTVYGRHNQPC : 250
 lllllllllllllllllllllllllll l
 260 *
Nl_ST64 : VRCGGLVVKETLGQRGTFYCPNCQK* : 275
Ng_FA1090 : LRCGGLVVKETLGQRGTFYCTNCQK* : 275
Nm_MC58 : PRCGGLVVKETLGQRGTFYCPNCQK* : 275
Nm_053442 : PQCGGLVVKETLGQRGTFYCPNCQK* : 275
Nm_Z2491 : PQCGGLVVKETLGQRGTFYCPNCQK* : 275
Nm_8013 : PQCGGLVVKETLGQRGTFYCPNCQK* : 275
Nm_FAM18 : PQCGGLVVKETLGQRGTFYCPNCQK* : 275
 lllll zinc fingerl ll

**Figure S1**

**Deduced amino acid sequence of Fpg homologues in Neisseria.**

Comparison of the deduced amino acid sequences of the Fpg homologue in 7 available *Neisseria* sequences. Essential motifs and catalytic residues, as well as the residues encoded by the DNA uptake sequence (DUS) are depicted. Colour coding is according to Table S1. Nl: *Neisseria* *lactamica*, Nm: *Neisseria* *meningitidis*, Ng: *Neisseria* *gonorrhoae*

* 20 * 40
Nm_MC58 : MPELPEVETTLRGIAPHIEGKTVEAV-VLRQLKLRWQI-NPDLGE : 43
E_coli : MPELPEVETSRRGIEPHLVGATILHA-VVRNGRLRWPV-SEEIYR : 43
L_lactis : MPELPEVETVRRELEKRIVGQKIVSIEATYPRMVLTGF--EQLKK : 43
Tt_HB8 : VPELPEVETTRRRLRPLVLGQTLRQV-VHRDP-ARYR--NTALAE : 41
B_stearoth : MPELPEVETIRRTLLPLIVGKTIEDVRIFWPNIIRHPRDSEAFAA : 45

 * 60 * 80 *
Nm_MC58 : ILSGRQVLSCGRRAKYLLIRFQTG-VLLIHLGMSGSLRIFTPSDG : 87
E_coli : -LSDQPVLSVQRRAKYLLLELPEG-WIIIHLGMSGSLRILPEELP : 86
L_lactis : ELTGKTIHGISRRGKYLIFEIGEKDRLISHLRMEGKYRLASLNVP : 88
Tt_HB8 : ---GRRILEVDRRGKFLLFALEGGVELVAHLGMTGGFRLEP---- : 79
B_stearoth : RMIGQTVRGLERRGKFLKFLLDRD-ALISHLRMEGRYAVASALEP : 89

 100 DUS * 120 *
Nm_MC58 : RIGRPDRHDHVDIVFSDGTVMRYRDPRKFGAILWY-EGIEEHHPL : 131
E_coli : ----PEKHDHVDLVMSNGKVLRYTDPRRFGAWLWT-KELEGHN-V : 125
L_lactis : ----MEKHDHLALKFTDEQLI-YADVRKFGTWELISTDQVLPYFL : 128
Tt_HB8 : -----TPHTRAALVL-EGRTLYFHDPRRFGRLFGVRRGDYREIPL : 118
B_stearoth : ----LEPHTHVVFCFTDGSELRYRDVRKFGTMHVYAKEEADRRPP : 130


 140 * 160 * 180
Nm_MC58 : LEKLGPEPLSEAFCADYLYARLKAQKRAVKLALMDNAVVVGVGNI : 176
E_coli : LTHLGPEPLSDDFNGEYLHQKCAKKKTAIKPWLMDNKLVVGVGNI : 170
L_lactis : KKNIGPEPTYETFDEQIFREKLQKSTKKIKPFLLEQTLVAGLGNI : 173
Tt_HB8 : LLRLGPEPLSEAFAFPGFFRGLKESARPLKALLLDQRLAAGVGNI : 163
B_stearoth : LAELGPEPLSPAFSPAVLAERAVKTKRSVKALLLDQTVVAGFGNI : 175
 lGPEPls F K L d v G GNI
 * * 220
Nm_MC58 : YANESLFRAGISPHRPANRLKKKECALLVETVKAVLQRAIETGGS : 221
E_coli : YASESLFAAGIHPDRLASSLSLAECELLARVIKAVLLRSIEQGGT : 215
L_lactis : YVDEVLWLAKIHPEKVANQLTESSIHLLHDSIIEILQKAIKLGGS : 218
Tt_HB8 : YADEALFRARLSPFRPARSLTEEEARRLYRALREVLAEAVELGGS : 208
B_stearoth : YVDESLFRAGILPGRPAASLSSKEIERLHEEMVATIGEAVMKGGS : 220
 Y E Lf A i P r A H2tH L l a GGs
 * 240 * 260 *
Nm_MC58 : TLRD--FVDSDGKSGYFQQEYTVYGRHNQPCPRCGGLVVKETLGQ : 264
E_coli : TLKD--FLQSDGKPGYFAQELQVYGRKGEPCRVCGTPIVATKHAQ : 258
L_lactis : SIRT---YSALGSTGKMQDELRVYGKTGEKCVRCGNEIQKIKVAG : 260
Tt_HB8 : TLSDQSYRQPDGLPGGFQTRHAVYGREGLPCPACGRPVERRVVAG : 253
B_stearoth : TVRT--YVNTQGEAGTFQHHLYVYGRQGNPCKRCGTPIEKTVVAG : 263
 t zinc finger a
 280
Nm_MC58 : RGTFYCPNCQK--- : 275
E_coli : RATFYCRQCQK--- : 269
L_lactis : RGTHFCPFCQQK-- : 272
Tt_HB8 : RGTHFCPTCQGEGP : 267
B_stearoth : RGTHYCPRCQR--- : 274
 RgT Cp C Q

**Figure S2**

**Deduced amino acid sequence of Fpg orthologues**.

The amino acid sequence of *N. meningitidis* MC58 Fpg compared to species for which the Fpg crystal structure has been solved. Essential motifs and catalytic residues, as well as the residues encoded by DNA uptake sequence (DUS) are depicted. Colour coding according to Table S1. Nm: *Neisseria* *meningitidis*; E. coli: *Escherichia* *coli*; L. lactis: *Lactococcus* *lactis*; Tt HB8: *Thermus* *thermophilus*; B. stearoth: *Bacillus* *stearothermophilus*

**Figure S3**

**Electrostatic charge of meningococcal Fpg.**

Distribution of the predicted electrostatic charge along the meningococcal Fpg amino acid sequence. Functional domains are illustrated; Fpg glycosylase domain (green), helix-2-turn-helix (H2tH) (red), Zinc finger (pink).

**Figure S4**

**Purified meningococcal Fpg.**

(A) Coomassie Brilliant Blue stained SDS-PAGE gel showing the recombinant expression and purification of *N. meningtidis* Fpg protein. Lane 1: SeeBlue® Plus2 Pre-Stained Standard, lane 2: *E. coli* whole cell lysate, lane 3: cleared lysate, lane 4: flow-trough, lanes 5 and 6: wash fractions, lanes 7-14: fractions eluted with 40, 60, 80, 100, 140, 180, 220, 250 mM Imidazole, respectively. Mc Fpg has an apparent size in SDS-PAGE of approximately 30 kDa, corresponding to the molecular weight predicted from the genome deduced amino acid sequence. (B) Coomassie Brilliant Blue stained SDS-PAGE gel showing the purified Fpg protein after dialysis. Lane 1:, SeeBlue® Plus2 Pre-Stained Standard, lane 2: the pooled elution fraction (100-140 mM Imidazole) that was used in the assays in this study.

**Figure S5**

**Meningococcal (Mc) Fpg activity on undamaged DNA substrate.**

Mc Fpg show no activity towards an undamaged double stranded DNA substrate after 1 hour incubation at 37^o^C, hence there is no Mc Fpg activity in the absence of 8oxoG residues. Lane 1: ladder, lane 2: double stranded DNA substrate, lane 2: double stranded DNA substrate incubated with 1 ng Mc Fpg, lane 3: double stranded DNA substrate incubated with 50 ng Mc Fpg.

References

1. Pereira de JK, Serre L, Hervouet N, Bouckson-Castaing V, Zelwer C, Castaing B: **Crystallization and preliminary X-ray crystallographic studies of a complex between the Lactococcus lactis Fpg DNA-repair enzyme and an abasic site containing DNA.** *Acta Crystallogr D Biol Crystallogr* 2002, **58:**679-682.

2. Saparbaev M, Sidorkina OM, Jurado J, Privezentzev CV, Greenberg MM, Laval J: **Repair of oxidized purines and damaged pyrimidines by E. coli Fpg protein: different roles of proline 2 and lysine 57 residues.** *Environ Mol Mutagen* 2002, **39:**10-17.

3. Sidorkina OM, Laval J: **Role of the N-terminal proline residue in the catalytic activities of the Escherichia coli Fpg protein.** *J Biol Chem* 2000, **275:**9924-9929.

4. Sugahara M, Mikawa T, Kumasaka T, Yamamoto M, Kato R, Fukuyama K, Inoue Y, Kuramitsu S: **Crystal structure of a repair enzyme of oxidatively damaged DNA, MutM (Fpg), from an extreme thermophile, *Thermus thermophilus* HB8.** *EMBO J* 2000, **19:**3857-3869.

5. Tchou J, Grollman AP: **The catalytic mechanism of Fpg protein. Evidence for a Schiff base intermediate and amino terminus localization of the catalytic site.** *J Biol Chem* 1995, **270:**11671-11677.

6. Tchou J, Bodepudi V, Shibutani S, Antoshechkin I, Miller J, Grollman AP, Johnson F: **Substrate specificity of Fpg protein. Recognition and cleavage of oxidatively damaged DNA.** *J Biol Chem* 1994, **269:**15318-15324.

7. Zharkov DO, Rieger RA, Iden CR, Grollman AP: **NH2-terminal proline acts as a nucleophile in the glycosylase/AP-lyase reaction catalyzed by Escherichia coli formamidopyrimidine-DNA glycosylase (Fpg) protein.** *J Biol Chem* 1997, **272:**5335-5341.

8. Gilboa R, Zharkov DO, Golan G, Fernandes AS, Gerchman SE, Matz E, Kycia JH, Grollman AP, Shoham G: **Structure of formamidopyrimidine-DNA glycosylase covalently complexed to DNA.** *J Biol Chem* 2002, **277:**19811-19816.

9. Amara P, Serre L, Castaing B, Thomas A: **Insights into the DNA repair process by the formamidopyrimidine-DNA glycosylase investigated by molecular dynamics.** *Protein Sci* 2004, **13:**2009-2021.

10. Amara P, Serre L: **Functional flexibility of Bacillus stearothermophilus formamidopyrimidine DNA-glycosylase.** *DNA Repair (Amst)* 2006, **5:**947-958.

11. Pereira de JK, Serre L, Zelwer C, Castaing B: **Structural insights into abasic site for Fpg specific binding and catalysis: comparative high-resolution crystallographic studies of Fpg bound to various models of abasic site analogues-containing DNA.** *Nucleic Acids Res* 2005, **33:**5936-5944.

12. Serre L, Pereira de JK, Boiteux S, Zelwer C, Castaing B: **Crystal structure of the *Lactococcus lactis* formamidopyrimidine-DNA glycosylase bound to an abasic site analogue-containing DNA.** *EMBO J* 2002, **21:**2854-2865.

13. Fromme JC, Verdine GL: **Structural insights into lesion recognition and repair by the bacterial 8-oxoguanine DNA glycosylase MutM.** *Nat Struct Biol* 2002, **9:**544-552.

14. Fromme JC, Verdine GL: **DNA lesion recognition by the bacterial repair enzyme MutM.** *J Biol Chem* 2003, **278:**51543-51548.

15. Lavrukhin OV, Lloyd RS: **Involvement of phylogenetically conserved acidic amino acid residues in catalysis by an oxidative DNA damage enzyme formamidopyrimidine glycosylase.** *Biochemistry (Mosc)* 2000, **39:**15266-15271.

16. Coste F, Ober M, Carell T, Boiteux S, Zelwer C, Castaing B: **Structural basis for the recognition of the FapydG lesion (2,6-diamino-4-hydroxy-5-formamidopyrimidine) by formamidopyrimidine-DNA glycosylase.** *J Biol Chem* 2004, **279:**44074-44083.

17. Perlow-Poehnelt RA, Zharkov DO, Grollman AP, Broyde S: **Substrate discrimination by formamidopyrimidine-DNA glycosylase: distinguishing interactions within the active site.** *Biochemistry (Mosc)* 2004, **43:**16092-16105.

18. Zaika EI, Perlow RA, Matz E, Broyde S, Gilboa R, Grollman AP, Zharkov DO: **Substrate discrimination by formamidopyrimidine-DNA glycosylase: a mutational analysis.** *J Biol Chem* 2004, **279:**4849-4861.

19. Sidorkina OM, Laval J: **Role of lysine-57 in the catalytic activities of Escherichia coli formamidopyrimidine-DNA glycosylase (Fpg protein).** *Nucleic Acids Res* 1998, **26:**5351-5357.

20. Rogacheva M, Ishchenko A, Saparbaev M, Kuznetsova S, Ogryzko V: **High resolution characterization of formamidopyrimidine-DNA glycosylase interaction with its substrate by chemical cross-linking and mass spectrometry using substrate analogs.** *J Biol Chem* 2006, **281:**32353-32365.

21. Bhagwat M, Gerlt JA: **3'- and 5'-strand cleavage reactions catalyzed by the Fpg protein from *Escherichia coli* occur via successive beta- and delta-elimination mechanisms, respectively.** *Biochemistry (Mosc)* 1996, **35:**659-665.

22. Harbut MB, Meador M, Dodson ML, Lloyd RS: **Modulation of the turnover of formamidopyrimidine DNA glycosylase.** *Biochemistry (Mosc)* 2006, **45:**7341-7346.

23. Zharkov DO, Shoham G, Grollman AP: **Structural characterization of the Fpg family of DNA glycosylases.** *DNA Repair (Amst)* 2003, **2:**839-862.

24. Castaing B, Geiger A, Seliger H, Nehls P, Laval J, Zelwer C, Boiteux S: **Cleavage and binding of a DNA fragment containing a single 8-oxoguanine by wild type and mutant FPG proteins.** *Nucleic Acids Res* 1993, **21:**2899-2905.

25. O'Connor TR, Graves RJ, de MG, Castaing B, Laval J: **Fpg protein of Escherichia coli is a zinc finger protein whose cysteine residues have a structural and/or functional role.** *J Biol Chem* 1993, **268:**9063-9070.

26. Tchou J, Michaels ML, Miller JH, Grollman AP: **Function of the zinc finger in Escherichia coli Fpg protein.** *J Biol Chem* 1993, **268:**26738-26744.

27. Rabow LE, Kow YW: **Mechanism of action of base release by Escherichia coli Fpg protein: role of lysine 155 in catalysis.** *Biochemistry (Mosc)* 1997, **36:**5084-5096.
